# Cost-consequence analysis of early full milk feeding versus gradual feeding with intravenous support in preterm infants: results from the FEED1 trial

**DOI:** 10.1136/archdischild-2025-329964

**Published:** 2025-12-21

**Authors:** Seyran Naghdi, Shalini Ojha, Jon Dorling, Josie Anderson, Chris Gale, Sophie Susannah Hall, Mark John Johnson, Samantha Johnson, Charlotte Kenyon, William McGuire, Garry Meakin, Eleanor Mitchell, Alan Montgomery, Sam J Oddie, Reuben Ogollah, Christopher Partlett, Yuanfei Su, Kate F Walker, Hema Mistry

**Affiliations:** 1 Warwick Clinical Trials Unit, University of Warwick, Coventry, UK; 2 Neonatal Unit, University Hospitals of Derby and Burton NHS Foundation Trust, Derby, UK; 3 Centre for Perinatal Research, University of Nottingham, Nottingham, Select, UK; 4 School of Medicine, University of Leeds, Leeds, England, UK; 5 Bliss, London, UK; 6 Academic Neonatal Medicine, Imperial College London, London, UK; 7 Nottingham Clinical Trials Unit, Applied Health Research Building, Nottingham, UK; 8 Human Development and Health, University of Southampton Faculty of Medicine, Southampton, UK; 9 Department of Neonatal Medicine, University Hospital Southampton NHS Foundation Trust, Southampton, UK; 10 Department of Population Health Sciences, University of Leicester, Leicester, Leicestershire, UK; 11 Centre for Reviews and Dissemination, University of York, York, North Yorkshire, UK; 12 Bradford Neonatology, Bradford Royal Infirmary, West Yorkshire, UK; 13 University Hospitals Coventry and Warwickshire, Coventry, UK

**Keywords:** Health Care Economics and Organizations, Neonatology

## Abstract

**Objective:**

To assess the economic consequences of initiating full milk feeds from birth compared with intravenous fluids with gradual feeding in infants born preterm.

**Design:**

Within-trial economic evaluation alongside a prospective, multicentre, randomised controlled trial (Fluids Exclusively Enteral from Day 1). A cost-consequence approach was used (revised from the planned cost-effectiveness analysis to avoid double counting length of stay within costs).

**Setting:**

46 UK National Health Service (NHS) neonatal units.

**Patients:**

Preterm infants born at 30+0to 32+6 weeks’ gestation.

**Interventions:**

Infants were allocated to either full milk feeds or gradual feeding with intravenous support within 3 hours of birth.

**Main outcome measure:**

Resource use and costs were captured from birth to 6 weeks’ corrected age. Costs were assessed from an NHS and personal social services perspective. The primary clinical outcome was length of hospital stay.

**Results:**

2088 infants were enrolled. There was no statistically significant difference in mean (95% CI) length of hospital stay between groups (−0.050 days (−0.638 to 0.538)). Mean total costs were £670 lower in the full milk group (95% CI: –£1562 to £223; p=0.141). Subgroup analyses suggested lower costs among infants born at 30 weeks’ gestation and those below the 10th birth weight centile; no evidence of interaction was found.

**Conclusions:**

Initiating full milk feeds from birth was associated with a modest reduction in costs compared with gradual feeding. While overall hospital stays and costs were not significantly reduced, early full feeding may offer economic advantages in selected subgroups. Further research is needed to assess long-term outcomes.

**Trial registration number:**

ISRCTN89654042.

**WHAT IS ALREADY KNOWN ON THIS TOPIC**

- Feeding practices for infants born preterm, at 30–32 weeks’ gestation, vary widely across neonatal units in the UK.
- Previous trials evaluating early feeding strategies have been small, underpowered and inconsistent in their design and definitions. Importantly, none of the existing trials investigating full enteral feeding have included a health economic evaluation.
- The Speed of Increasing Milk Feeds Trial, conducted in very preterm or very low birth weight infants, compared faster versus slower daily feed increments, found no overall cost-effectiveness benefit and raised concerns about potential harm associated with a faster feeding strategy, although this was not reflected in the primary outcome.
- No trial has yet evaluated the clinical and economic impact of initiating full milk feeds from birth in preterm infants born between 30+0 weeks’ and 32+6 weeks’ gestation.

**WHAT THIS STUDY ADDS**

- Fluids Exclusively Enteral from Day 1 (FEED1) is the first within-trial economic evaluation to assess the impact of initiating full milk feeds from birth in preterm infants born between 30+0 weeks’ and 32+6 weeks’ gestation.
- This study provides direct evidence from a randomised controlled trial on whether full milk feeding from day one offers a cost-consequence advantage over standard gradual feeding supported by intravenous fluids or parenteral nutrition.

**HOW THIS STUDY MIGHT AFFECT RESEARCH, PRACTICE OR POLICY**

- Initiating full milk feeds from birth in preterm infants may shift care from intensive to lower-dependency settings. These findings are relevant in the context of health service pressures and workforce shortages. While the cost reduction was modest, the impact on cot availability (for the scarcest intensive-care cots), maternity bed availability and impact on staffing levels (for the most highly staffed levels of care) is likely to have cost savings not captured by this analysis. While a full cost-effectiveness analysis was not conducted, the cost-consequence approach provides timely evidence to inform neonatal care planning and policy.

## Introduction

 In preterm infants, early nutrition plays a vital role in promoting gut maturation, supporting growth and reducing complications.^1^ Yet, feeding practices vary widely across neonatal units.^2^ In high-income settings, almost all babies born at 30–32 weeks’ gestation will be initially managed with intravenous fluids or parenteral nutrition, while milk feeds are introduced gradually over several days.^3^ This cautious approach reflects concerns about feeding intolerance and the risk of necrotising enterocolitis (NEC). However, NEC is uncommon in this group, affecting fewer than 1% of infants born after 30 weeks of gestation.^4^

Recent randomised controlled trials (RCTs) in preterm infants suggest that starting milk feeds earlier or advancing them more quickly can lower the risk of invasive infection, without increasing rates of NEC or mortality.^5^ In preterm infants, earlier full enteral feeding might improve weight gain, reduce reliance on intravenous fluids and central lines and support earlier hospital discharge.^6^

These benefits are particularly relevant given ongoing pressures in the UK health service. Infants born at 30–32 weeks’ gestation typically require three to 6 weeks of inpatient care, with each additional day in hospital costing the National Health Service (NHS) an estimated £800–£1200, depending on the level of care.^7^ Use of parenteral nutrition and central venous catheters is associated not only with higher direct costs but also an increased risk of catheter-related bloodstream infection, with each episode estimated to add £10 000–£15 000 in treatment and extended hospitalisation.^8^ Prolonged stays also place emotional and financial strain on families, reduce cot availability and increase staffing demands in neonatal units already facing workforce shortages.^9 10^

Despite these considerations, robust evidence on the clinical and economic impact of initiating full milk feeds from birth in preterm infants is lacking.^11^ Previous trials have been small, underpowered and inconsistent in their definitions and implementation of early feeding strategies.^12^ To address this, the Fluids Exclusively Enteral from Day 1 (FEED1) trial assessed whether initiating full milk feeds within 3 hours of birth in infants born between 30+0 weeks’ and 32+6 weeks’ gestation reduced the duration of hospital stay compared with intravenous fluids with gradual feeding.^11^ The FEED1 trial found that full milk feeding from birth was feasible and safe but did not reduce the overall length of hospital stay compared with gradual feeding.^13^ This paper reports the cost-consequence analysis undertaken alongside FEED1.

## Methods

### Trial background

The FEED1 trial was a prospective, multicentre, RCT. Details of the trial have been published elsewhere.^11^ Briefly, infants born between 30+0 weeks’ and 32+6 weeks’ gestation were eligible if they were less than 3 hours old, clinically stable and without congenital abnormalities affecting gastrointestinal function.^11^ Eligible infants at 46 UK NHS neonatal units were enrolled in the trial and allocated either to receive full milk feeds from birth or gradual feeding supported by intravenous fluids or parenteral nutrition. The primary clinical outcome was length of neonatal hospital stay.^11^

### Overview of economic analyses

The economic evaluation aimed to compare the cost and outcomes associated with full milk feeds vs intravenous fluids with gradual feeding for preterm infants based on data collected during the RCT.^11^ A cost-consequence analysis was undertaken, and an NHS and personal social services (PSS) perspective was adopted.^14^ The time horizon covered the period from randomisation to 6 weeks’ corrected age. No discounting was applied. The analysis was carried out in accordance with the Health Economics Analysis Plan (HEAP).

### Diversion from HEAP

Although the HEAP specified a cost-effectiveness analysis with length of hospital stay as the primary outcome, this approach was reconsidered during the analysis stage. Since the length of hospital stay is a key driver of cost and is fully reflected in the resource use and cost data, using it as the effectiveness measure in an incremental cost-effectiveness ratio would result in methodological overlap by double counting and potentially any conclusions made can be misleading.^15^ To address this issue, a cost-consequence approach was adopted, allowing costs and outcomes to be reported separately.^15^ All other analytical procedures continued to follow the HEAP.

### Measurement of resource use and costs

Data on the health service resources used to treat each infant between randomisation and initial hospital discharge were collected and reported on case record forms. This included the type and duration of the infant(s) stay in hospital differentiated by level of care (intensive care, high dependency or special care), whether the infant was transferred to another hospital, any neonatal procedures or tests which were undertaken, any adverse events which may have happened and medications which were administered.

Parent-completed questionnaires were used to collect resource use data for infants from the time of initial hospital discharge to 6 weeks’ corrected age. This included hospital readmissions, outpatient visits, accident and emergency attendances, medication use, formula feeding and use of community health and social care services.

Length of hospital stay was capped at a maximum of 112 days since birth, reflecting the time required to reach 6 weeks’ corrected age for infants born at 30 weeks’ gestation. This corresponds to 10 weeks to reach full term (40 weeks’ gestation) plus 6 weeks corrected age (ie, post-term equivalent age).

### Valuation of resource use

Resource use was valued using unit costs sourced from national databases (online supplemental appendix 1). We used the National Schedule of NHS costs for 2023/2024 to assign values to infants’ hospital admissions and outpatient visits.^16^ For inpatient stays, Healthcare Resource Group (HRG) codes were used. HRG tariffs for neonatal critical care bundles included the cost of routine medications, except for any high-cost drugs. Any high-cost drugs used have been costed separately in line with NHS tariff policy.^17 18^ The costs associated with community-based health and social care services were determined using unit cost data from the Unit Costs of Health and Social Care 2023 compendium.^19^ The cost of medications for each infant was derived from the Children’s British National Formulary and the Prescription Cost Analysis databases.^20 21^ For each medication, the cost was computed based on the dosages and frequencies that were provided. All costs were in pounds sterling for 2023/2024. Any unit costs not in this price year were adjusted to the 2023/2024 price levels.^22^

We used multilevel Poisson or negative binomial regression models to compare healthcare resource use between treatment groups. The model used depended on whether the data showed greater variability than expected (known as overdispersion). If the variability was high (Pearson χ² statistic divided by df >1.5), we used the negative binomial model instead of the Poisson model. Models were adjusted for gestational age, birth weight centile and intravenous fluid use, with random effects specified at both the site and mother levels to account for multiple births. SEs were provided for the means of the treatment groups, and bootstrap-generated 95% CIs were presented for the differences in mean resource use and cost estimates between the groups.

### Measurement of outcomes

The primary outcome was the reduction in the number of days in hospital care (including intensive care, high dependency or special care), measured from birth to discharge. This matches the primary clinical outcome of the FEED1 trial and is summarised using mean values with associated standard errors for each treatment group. Differences between groups were assessed using multilevel generalised linear models (same as resource use analysis).

### Handling of missing data

Any missing costs and length of hospital stay data were handled using a fully conditional multiple imputation technique, implemented via chained equations (MICE) in Stata V.18.0.^23^ The imputation procedure was conducted under the assumption that the missing data occurred at random.^24^ We then used logistic regression models to investigate whether initial admission characteristics predicted the likelihood of missing data.

Covariates used to predict missing values included gestational age, birth weight centile, treatment allocation, intravenous fluid use, site, mother identifier to reflect multiple birth effects, sex and ethnicity, along with the outcome variables (total mean cost and length of stay) to preserve associations between predictors and outcomes. Imputations were conducted separately for each group using predictive mean matching, selecting the five closest observed values for each missing case and the process ran 50 times. Patterns of missingness were summarised descriptively, and no statistical tests were performed to compare missing-data proportions between treatment groups.

### Base-case analysis

The base-case analysis used a modified intention-to-treat (modified-ITT) approach with imputed data and applied a 112-day cap on length of hospital stay to reflect the trial’s maximum intended follow-up period. The modified-ITT population excluded those infants who had missing data on the primary outcome due to death before hospital discharge or withdrawal of consent. A non-parametric bootstrap method was applied to estimate uncertainty around the mean differences in total costs and length of hospital stay between the groups. A total of 1000 bootstrap samples were drawn, and for each replicate, we calculated mean estimates and 95% CIs for differences in total costs and length of hospital stay between groups. Rubin’s rules were used to combine estimates across the 50 imputed datasets, accounting for both withinimputation and between-imputation variability.^25^ We validated the imputation model by comparing the distributions of imputed and observed values to ensure plausibility. Data analysts were blind to treatment allocation until after database lock.

### Sensitivity and subgroup analysis

Sensitivity analyses were conducted to assess the robustness of the results:

1. Complete cases only, using infants with fully observed cost and outcome data, applying the 112-day cap.
2. Complete case analysis, using infants with fully observed cost and outcome data, without applying the 112-day cap (using all observed follow-up data).
3. Imputed data analysis using the modified-ITT population, without the 112-day cap.
4. A full ITT analysis, including all randomised infants (including those who died or withdrew consent), applying the 112-day cap on length of hospital stay.

Prespecified subgroup analyses were conducted to explore variation in costs and outcomes by:

1. Week of gestational age at birth (30, 31 and 32).
2. Birth weight centile adjusted for gestational age (<10^th^ centile vs ≥10^th^ centile).

### Post-hoc analysis

Following the main analysis, we identified that parent-reported readmissions to intensive care following initial discharge appeared implausibly high. The cause of this discrepancy is unclear, but it may reflect misreporting or misinterpretation by parents, potentially due to confusion between readmission for paediatric intensive care after discharge and the infant’s initial stay in neonatal intensive care immediately after birth. To address this, we conducted a post-hoc analysis excluding the potentially inaccurate postdischarge intensive care data.

## Results

### Missing data

For length of hospital stay, missing data were very low and balanced between groups, with 1.7% missing in both groups. Resource-use data up to discharge were also largely complete, with 2.1% missing in the full milk group and 1.9% in the gradual feeding group (2.0% overall). Missing data were higher in the gradual feeding group across all resource use categories at 6 weeks’ corrected age (51.1% in the full milk group and 55.2% in the gradual feeding group) (online supplemental table S1).

### Health and social care resource use

Significant differences in resource use for complete cases only were identified across several inpatient categories (table 1). Infants in the full milk feeding group had a significantly shorter mean neonatal intensive care stays (MD= –1.6 days, 95% CI: –2.4 to –0.8, p<0.001) compared with those in the gradual feeding group. However, they experienced a longer stay in neonatal special care (MD=1.8 days, 95% CI 0.5 to 3.0, p=0.005). No significant differences were seen in neonatal high dependency care (MD= –0.2 days, p=0.631) or in transitional care (MD= –0.4 days, p=0.194).

**Table 1:** Use of health and social care resources treatment group (complete case)

Resource	Full milk feeding (mean, SE) (n=1029)	Gradual feeding (mean, SE) (n=1023)	Mean difference (95% CI)	P value
Infants with complete data	n=512	n=465		
**Inpatient care (from randomisation to initial discharge**)				
Neonatal intensive care (days)	1.3 (0.3)	2.9 (0.6)	−1.6 (−2.4 to −0.8)	<0.001
Neonatal high dependency care (days)	5.1 (0.4)	5.3 (0.5)	−0.2 (−1.0 to 0.6)	0.631
Neonatal special care (days)	26.0 (0.7)	24.2 (0. 7)	1.8 (0.5 to 3.0)	0.005
Special care with primary carer resident or transitional care (days)	2.3 (0.7)	2.8 (0.9)	−0.4 (–1.1 to 0.2)	0.194
Total number of neonatal critical care (days)	33.3 (0.6)	32.3 (0.5)	1.0 (−0.5 to 2.6)	0.216
Transfer to different hospital (count)	0.393 (0.045)	0.320 (0.039)	0.072 (–0.010 to 0.155)	0.087
**6 weeks corrected age**				
**Community healthcare (number of contacts**)				
GP visit at surgery	1.619 (0.069)	1.584 (0.071)	0.034 (−0.143 to 0.212)	0.703
GP visit at home	0.140 (0.049)	0.032 (0.014)	0.108 (0.007 to 0.209)	0.036
GP phone	1.419 (0.092)	1.303 (0.088)	0.116 (−0.132 to 0.364)	0.360
Practice nurse	1.238 (0.088)	1.245 (0.090)	−0.007 (−0.149 to 0.135)	0.921
Health visitor at surgery	0.644 (0.081)	0.685 (0.094)	−0.041 (−0.197 to 0.115)	0.604
Health visitor at home	3.182 (0.228)	3.035 (0.220)	0.147 (−0.137 to 0.430)	0.310
Neonatal nurse at home	2.994 (0.519)	2.920 (0.504)	0.074 (−0.380 to 0.528)	0.749
Walk-in centre	0.389 (0.078)	0.327 (0.055)	0.062 (−0.114 to 0.238)	0.488
Other health professionals	0.424 (0.073)	0.223 (0.042)	0.201 (0.056 to 0.345)	0.006
**Inpatient care from initial discharge to 6 weeks corrected age**				
Intensive care unit ward (days)	2.2 (0.5)	3.1 (0.8)	−0.9 (−2.8 to 0.9)	0.329
Paediatric ward (days)	1.2 (0.3)	0.6 (0.1)	0.6 (−0.1 to 1.3)	0.086
Day case (days)	0.4 (0.1)	0.5 (0.1)	−0.1 (−0.3 to 0.1)	0.275
**Outpatient care (number of visits**)				
Outpatient clinic	1.207 (0.085)	1.167 (0.085)	0.040 (−0.153 to 0.233)	0.685
Accident and emergency department	0.398 (0.042)	0.395 (0.043)	0.003 (−0.104 to 0.110)	0.956
Other health professionals	0.424 (0.073)	0.223 (0.042)	0.201 (0.056 to 0.345)	0.006

GP, general practice.

Among community-based resources, infants in the full milk group, participants had significantly more GP home visits (MD 0.108 visits, 95% CI 0.007 to 0.209, p=0.036) and had more contacts with other health professionals (MD 0.201 visits, 95% CI 0.056 to 0.345, p=0.006).

### Economic costs

From randomisation to initial discharge, neonatal intensive care costs were significantly lower for the full milk feeding group compared with gradual feeding (MD −£1539.84, 95% CI −£2915.31 to −£164.36, p=0.028) (table 2). However, neonatal special care costs were significantly higher for the full milk group (MD £2251.42, 95% CI £825.54 to £3677.30, p=0.002). No significant differences were found in neonatal high dependency care, transitional care or hospital transfer costs.

**Table 2:** Full milk feeding versus gradual feeding; NHS and PSS costs for complete case, study period and cost category (£, 2023–2024 prices)

Cost description*	Treatment group, mean adjusted† (SE) cost (£)		Between-group differences (95% CI)	P value
	Full milk feeding (n=1029)	Gradual feeding (n=1023)		
Infants with complete data	n=512	n=465		
**Initial admission** (**from randomisation to initial discharge**)				
Neonatal intensive care (days)	2420.31 (430.98)	3960.15 (705.55)	−1539.84 (−2915.31 to −164.36)	0.028
Neonatal high dependency care (days)	7144.19 (728.00)	7563.05 (791.29)	−418.85 (−2.267.22 to 1429.51)	0.657
Neonatal special care (days)	27 979.18 (770.56)	25 727.75 (726.60)	2251.42 (825.54 to 3677.30)	0.002
Special care with primary carer resident or transitional care	1115.95 (93.11)	1389.99 (122.71)	−274.03 (−578.02 to 29.95)	0.077
Transfer to different hospital	652.19 (99.20)	529.18 (79.28)	123.01 (−115.97 to 361.99)	0.313
Antibiotic‡	0.03 (0.00)	0.03 (0.00)	0.00 (−0.01 to 0.01)	0.991
**Total initial admission cost**	**38 509.87** (**782.70**)	**39 253.16** (**816.83**)	**−743.29 (−2379.42 to 892.84**)	**0.373**
**6 weeks’ corrected age**				
GP visit (surgery)	79.48 (4.92)	77.47 (5.02)	2.01 (−11.78 to 15.80)	0.775
GP visit (home)	0.09 (0.01)	0.07 (0.01)	0.02 (0.00 to 0.04)	0.033
GP phone	69.94 (6.98)	64.38 (6.75)	5.56 (−13.55 to 24.67)	0.570
Practice nurse	14.91 (1.19)	14.99 (1.26)	−0.09 (−3.47 to 3.29)	0.960
Health visitor at surgery	7.07 (0.59)	7.57 (0.67)	−0.50 (−2.24 to 1.24)	0.573
Neonatal nurse (home)	81.88 (16.68)	76.56 (14.84)	5.32 (−16.83 to 27.47)	0.638
Health visitor (home)	72.91 (2.55)	67.94 (2.49)	4.97 (−2.01 to 11.95)	0.163
Walk-in centre	65.99 (9.90)	57.65 (9.33)	8.34 (−18.61 to 35.30)	0.544
Visit of any other health professionals community based	20.62 (5.73)	35.04 (10.17)	−14.42 (−37.42 to 8.58)	0.219
**Community cost**	**417.29** (**15.84**)	**394.25** (**15.65**)	**23.04 (−16.90 to 62.98**)	**0.258**
Intensive care unit ward	2282.81 (414.96)	3262.37 (662.26)	−979.56 (−2441.21 to 482.08)	0.189
Paediatric ward	529.80 (125.24)	244.59 (59.42)	285.22 (16.55 to 553.89)	0.037
Day case	277.91 (39.25)	299.06 (44.26)	−21.15 (−135.91 to 93.61)	0.718
Outpatient clinic	417.83 (23.34)	414.94 (24.39)	2.90 (−62.22 to 68.02)	0.930
Accident and emergency department	88.47 (8.28)	93.89 (9.22)	−5.42 (−29.65 to 18.81)	0.661
Visit of any other health professionals hospital based	102.28 (21.39)	60.34 (13.53)	41.93 (−7.07 to 90.93)	0.094
Procedures	17.50 (12.89)	5.10 (2.69)	12.40 (−12.40 to 37.21)	0.327
**Total hospital cost**	**3816.16** (**545.33**)	**4035.69** (**591.52**)	**−219.52 (−1504.53 to** 1065.48)	**0.738**
Formula	125.77 (17.26)	142.39 (19.89)	−16.62 (−59.11 to 25.87)	0.443
Medications	20.90 (1.89)	21.73 (2.02)	−0.83 (−5.84 to 4.18)	0.745
**6-week follow-up**	**3702.68** (**316.93**)	**3770.07** (**332.07**)	**−67.39 (−746.38 to 611.60**)	**0.846**
**Total cost**	**42 781.50** (**861.45**)	**43 619.54** (**905.46**)	**−838.04 (−2829.97 to** 1153.89)	**0.410**

* Healthcare costs are capped and reported with a maximum of 112 days (reflecting the study follow-up period).

† Adjusted for gestational age, birth weight centile and intravenous fluids; random effects account for clustering by site and mother (to capture multiple births).

‡ Only includes high price drugs which were not covered by critical care Healthcare Resource Group codes.

NHS, National Health Service; PPS, personal social service.

At 6 weeks’ corrected age, no significant differences were found between groups in the total community costs or with the individual resource use components. However, there were slightly more GP home visit costs in the full milk feeding group (MD=£0.02, p=0.033). Costs related to paediatric ward stays were also significantly higher in this group (MD=£285.22, p=0.037).

The overall adjusted total cost combining the randomisation to initial discharge costs and 6-week follow-up care costs was slightly lower and not statistically significant for the full milk feeding group (MD −£838.04, 95% CI −£2829.97 to £1153.89, p=0.410).

### Health outcomes

Among infants with complete data (length of stay capped at 112 days), the adjusted mean length of hospital stay was 32.2 days in the full milk group (n=512) and 32.1 days in the gradual feeding group (n=465) before discharge. The between-group difference was not statistically significant at 0.1 days (95% CI –1.2 to 1.4; p=0.875), equivalent to approximately 2.4 hours.

### Cost-consequences results

#### Base-case analysis

The base-case analysis used imputed data with a modified-ITT approach (excluding 36 infants who died or whose parents withdrew consent) and applied a 112-day cap on hospital stay (table 3). Only four infants exceeded 112 days (three in the full milk feeding group and one in the gradual feeding group). The mean NHS and PSS cost was slightly lower in the full milk feeding group with an adjusted mean difference of –£669.78 (95% CI –£1562.18 to £222.62, p=0.141). Mean length of hospital stay was 32.0 days in the full milk group and 32.1 days in the gradual feeding group, with a negligible adjusted difference of –0.1 days (95% CI –0.6 to 0.5, p=0.867).

**Table 3:** Cost-consequences results (£, 2023–2024 prices); comparing full milk feeding from day one with gradual feeding

Scenario	Treatment group, mean (SE) cost (£)				Incremental cost (95% CI)	P value	Treatment group, mean (SE) reduction of days in hospital care		Differences in length of hospital stay (day; 95% CI)	P value
	N	Full milk feeding	N	Gradual feeding			Full milk feeding	Gradual feeding		
Base case analysis										
Imputed cost and observed length of hospital stay; covariate adjusted (modify ITT)*	1029	42 824.94 (786.37)	1023	43 494.72 (798.80)	−669.78 (−1562.18 to 222.62)	0.141	32.0 (0.6)	32.1 (0.6)	−0.1 (−0.6 to 0.5)	0.867
Sensitivity analysis										
Complete case analysis of cost and length of hospital stay; covariate-adjusted	512	42 781.50 (861.45)	465	43 619.54 (905.46)	−838.04 (−2353.96 to 677.88)	0.279	32.2 (0.6)	32.1 (0.7)	0.1 (−0.9 to 1.1)	0.838
Imputed cost and observed length of hospital stay; covariate-adjusted -all observed follow-up data† (modify ITT*)	1029	43 040.67 (795.35)	1023	43 662.50 (810.20)	−621.83 (−1552.84 to 309.17)	0.191	32.1 (0.6)	32.1 (0.6)	−0.1 (−0.7 to 0.5)	0.814
Complete case analysis of cost and length of hospital stay; covariate-adjusted†	512	42 950.44 (872.97)	465	43 795.68 (918.31)	−845.24 (−2399.22 to 708.74)	0.286	32.3 (0.6)	32.2 (0.7)	0.7 (−0.9 to 1.1)	0.905
Imputed cost and length of hospital stay; covariate adjusted (ITT)	1047	42 899.50 (789.28)	1041	43 612.44 (820.81)	−712.94 (−1586.94 to 161.06)	0.110	32.1 (0.6)	32.2 (0.6)	−0.1 (−0.6 to 0.5)	0.827
Post-hoc analysis										
Imputed cost and observed length of hospital stay; covariate adjusted (modify ITT)*; excluding neonatal intensive care readmission data	1029	40 363.52 (710.48)	1023	40 752.84 (715.33)	−389.32 (−1152.53 to 373.90)	0.317	32.0 (0.6)	32.1 (0.6)	−0.1 (−0.6 to 0.5)	0.865
Complete case analysis of cost and length of hospital stay; covariate-adjusted; excluding neonatal intensive care readmission data	512	40 266.44 (774.77)	465	40 875.73 (810.22)	−609.29 (−1818.65 to 600.07)	0.323	32.1 (0.6)	32.1 (0.7)	0.0 (−0.9 to 0.9)	0.991
Subgroup analysis										
30 weeks of gestation at birth	229	57 106.42 (1623.01)	233	61 113.52 (1850.07)	−4026.10 (−6419.74 to −1632.47)	0.001	43.1 (1.0)	42.6 (1.0)	0.4 (−1.0 to 1.9)	0.557
31 weeks of gestation at birth	348	44 325.00 (1080.31)	345	44 736.00 (1134.74)	−411.00 (−2067.44 to 1245.44)	0.627	33.6 (0.7)	33.4 (0.7)	0.3 (−0.8 to 1.4)	0.626
32 weeks of gestation at birth	470	34 404.86 (904.40)	463	34 165.27 (890.02)	239.59 (−948.53 to 1427.71)	0.693	25.4 (0.6)	25.7 (0.6)	−0.2 (−1.1 to 0.6)	0.561
Birth weight centile <10	126	47 788.73 (1893.53)	116	51 874.87 (2101.41)	−4,086.15 (−7033.91 to −1138.39)	0.007	36.8 (1.1)	38.4 (1.2)	−1.7 (−4.3 to 1.0)	0.216
Birth weight centile ≥10	921	42 471.20 (807.90)	925	42 658.43 (813.10)	−187.23 (−1122.28 to 747.81)	0.695	31.5 (0.6)	31.7 (0.6)	0.2 (−0.5 to 0.9)	0.544

* 36 infants who died or withdrew consent before discharge were excluded from the analysis, and no imputation was performed for these cases.

† All cost and resource use outcomes are based on the full observed follow-up period for each infant. No 112-day cap was applied, allowing the analysis to reflect the complete resource use and costs as recorded during the study.

ITT, intention to treat.

#### Sensitivity and subgroup analyses

Sensitivity analyses supported the base-case findings (table 3). Subgroup analyses by gestational age and birth weight centile suggested potential variation in cost outcomes. Among 462 infants born at 30 weeks, full milk feeding was associated with significantly lower costs (MD –£4026.10; 95% CI –£6419.74 to –£1632.47; p=0.001). However, among infants born at 32 weeks, those in the full milk group had a non-significant slight increase in cost (MD £239.59; 95% CI –£948.53 to £1427.71; p=0.693). No significant differences were found in length of hospital stay across any gestational age groups. Among 242 infants born <10^th^ birth weight centile, full milk feeding was associated with a lower cost (MD –£4086.15, 95% CI –£7033.91 to –£1138.39, p=0.007) and a shorter mean hospital stay (MD –1.7 days, 95% CI –4.3 to 1.0, p=0.216). Formal interaction testing showed no evidence that treatment effects differed by gestational age or birth weight centile (gestational age interaction: χ²(2)=2.61, p=0.27; birth weight centile interaction: χ²(1)=1.54, p=0.21).

#### Post-hoc analysis

The post-hoc analysis showed results that remained consistent in direction and magnitude with the primary analysis. The observed differences in costs and hospital stay were small and statistically non-significant, indicating that the discrepancy had minimal impact on the overall findings (table 3; (online supplemental appendix A2).

## Discussion

The economic evaluation found that full milk feeding was associated with a modest reduction in costs compared with gradual feeding, although the differences were not statistically significant. The base-case modified-ITT analysis showed a mean cost difference of –£670, with no meaningful difference in length of hospital stay (–0.1 days; ~72 min). These results were consistent across sensitivity analyses.

In our subgroup analyses, infants born at 30 weeks had lower mean costs in the full milk feeding (~£4000 difference) despite similar lengths of hospital stay, and infants below the 10^th^ birth weight centile also showed lower mean costs. However, formal interaction testing showed no evidence that treatment effects differed by gestational age or birth weight centile, indicating these findings should be interpreted with caution.

A possible explanation for the absence of cost savings despite earlier full feeding lies in the type rather than duration of care. Infants in the full milk group had shorter stays in neonatal intensive care but more days receiving special care, which are typically lower-cost settings. As such, resource use shifted, but the total duration of hospital stay remained the same. Discharge timing is typically determined by achieving full oral feeding, adequate weight gain and physiological stability rather than the level of care, so earlier transfer to lower-dependency care does not necessarily shorten the overall hospital stay.^26^ This interpretation aligns with existing evidence. Morgan *et al* reported that early enteral feeding in preterm infants may support gastrointestinal maturity and clinical stability without necessarily shortening hospital stays.^12^ Similarly, Groff *et al* noted that early enteral feeding promotes neurodevelopment and immune function, yet consistent reductions in discharge times have not been observed.^27^

These findings are consistent with previous evidence. The SIFT trial also reported no overall cost-benefit from a faster daily incremental feeding strategy in very preterm infants.^25^ However, two recent RCTs have reported that earlier or exclusive enteral feeding may reduce hospital stay, one in infants 30–33 weeks’ gestation and another in very preterm infants.^28 29^ If early feeding reduces length of stay, as reported in recent RCTs, our cost estimates suggest that each inpatient day avoided would save approximately £1200–£1300 per infant.

We noted that there were no differences in hospital readmissions or outpatient contacts at follow-up. Although there appeared to be more GP or other health professionals’ contacts in the full milk group, this should be interpreted cautiously. The finding may reflect random variation or loss to follow-up due to substantial missing postdischarge data rather than a true difference in service use. Taken together, these findings suggest that initiating full milk feeding from day one is associated with a modest reduction in costs compared with gradual feeding, with potential for cost savings in certain subgroups. While full milk feeding did not reduce the length of hospital stay for these infants, it did not lead to any further increased costs or adverse economic outcomes.

### Strengths and limitations

This evaluation benefits from being embedded in a large, multicentre RCT, which allowed for high-quality data collection across a diverse population. The use of multiple imputation methods helped address missing data, and planned subgroup and sensitivity analyses provided a broader understanding of potential variation in outcomes. However, missing data were high for 6-week measures, and while imputation helps to mitigate this, it cannot fully eliminate the risk of bias.

The study’s follow-up was limited to 6 weeks’ corrected age, meaning it does not reflect possible longer-term differences in health outcomes, development of these infants or service use. The analysis also focused solely on healthcare and social care costs, without incorporating quality of life measures or wider family and societal impacts.

We were not able to differentiate the reasons for delayed discharge or compare expected vs actual discharge dates. Detailed clinical causes of delayed discharge were not captured in the economic dataset and therefore could not be linked to cost patterns.

## Conclusion

Providing full milk feeds from the first day of life in preterm infants was associated with a modest reduction in healthcare costs compared with gradual feeding supported by intravenous fluids. Although overall differences in hospital stay and costs were not statistically significant, subgroup analyses suggest potential savings in certain groups, including infants born below the 10^th^ birth weight centile and those born at 30 weeks’ gestation; but no evidence of interaction was found. The pattern of resource use also indicates that early feeding may reduce the need for intensive care, with care potentially shifting to lower-dependency settings. This may reflect a more efficient use of healthcare resources. Further research is needed to assess longer-term outcomes and costs beyond 6 weeks’ corrected age.

## Supplementary material

10.1136/archdischild-2025-329964 online supplemental file 1

10.1136/archdischild-2025-329964 online supplemental file 2

## Data Availability

Data are available upon reasonable request.
