# Supplementary material for: Cost-consequence analysis of early full milk feeding versus gradual feeding with intravenous support in preterm infants: results from the FEED1 trial
Source: Arch Dis Child Fetal Neonatal Ed. 2025 Dec 21;111(3):e329964. doi: 10.1136/archdischild-2025-329964 (PMC13151484; doi:10.1136/archdischild-2025-329964)
Supplement: online supplemental file 1 [file fetalneonatal-111-3-s001.pdf]

Table S1: Number and proportion of individuals with missing health economic data by treatment group

Description	Treatment group, missing values, n (%)		Total missing values, n (%)
	Full milk feeding (n=1,047)	Gradual feeding (n=1,041)	
**Primary Outcome**			
Length of hospital stay	18 (1.72)	18 (1.73)	36 (1.72)
**Resources used from initial admission to discharge**			
Hospital admission	22 (2.10)	20 (1.92)	44 (2.01)
**Resources used at six weeks corrected age**			
Community-based health and social care services	386 (36.87)	425 (40.83)	811 (38.84)
Hospital-based services	378 (36.10)	412 (39.87)	790 (37.84)
Formula	452 (43.17)	507 (48.70)	959 (45.93)
Medications	454 (43.36)	493 (47.36)	947 (45.35)
Total resource use missing at six weeks' corrected age	535 (51.10)	575 (55.24)	1,110 (53.16)
