# Supplementary material for: Cost-consequence analysis of early full milk feeding versus gradual feeding with intravenous support in preterm infants: results from the FEED1 trial
Source: Arch Dis Child Fetal Neonatal Ed. 2025 Dec 21;111(3):e329964. doi: 10.1136/archdischild-2025-329964 (PMC13151484; doi:10.1136/archdischild-2025-329964)
Supplement: online supplemental file 2 [file fetalneonatal-111-3-s002.pdf]

Appendix 1

Table A1: Unit costs associated with infant's resource use items

Community care; Source: Unit Costs of Health and Social Care 2023/2024[17]				
**HRG code**	**Currency description**	**Unit cost (£)**	**Ordinary trim-point**	**Unit cost for days exceeding time-point (£)**
PX30C	Paediatric Admission for Unexplained Symptoms, with CC Score 0	700	5	486
**Type of appointment**	**Description**	**Unit Cost per hour (£)**	**Unit cost per visit (£)**	
GP visit at home	Per hour of patient contact including travel time	296	148	
GP telephone calls	Average cost per e-consultation	-	14.43	
GP surgery visit	Per surgery consultation lasting 9.22 minutes	-	49	
Practice nurse	15 minutes of practice nurse at surgery	47	11.75	
Health visitor at surgery	15 minutes of band 4 qualified nurse	44	11	
Health visitor at home	30 minutes of band 4 qualified nurse including travel time	44	22	
Neonatal nurse at home	30 minutes of band 5 qualified nurse at home	53	26.5	
Eye consultant	15 minutes of a medical consultant	143	35.75	
Midwife	15 minutes of band 6 qualified nurse at home	64	16	
Neonatal consultant	15 minutes of neonatal consultant	144.67	36.17	
Physiotherapy	30 minutes of band 7 Physiotherapist	63	31.5	
Paediatrics	15 minutes of paediatrics consultant	162.91	40.73	
Speech and language therapy	15 minutes of band 7 speech and language therapist	63	15.75	
Pharmacy	15 minutes of band 6 pharmacist	53	13.25	
Dietician	15 minutes of dietician	50.64	12.66	
Walk-in-centre	30 minutes of NHS walk-in centres; emergency medicine, category 1 investigation with category 1-2 treatment	173	86.5	
Inpatient care; Source: National schedule of NHS costs - Year 2023/24[14]				
**HRG code**	**Currency description**	**Unit cost per day (£)**		
XA01Z	Neonatal Critical Care, Intensive Care	2,127		
XA02Z	Neonatal Critical Care, High Dependency	1,483		
XA03Z	Neonatal Critical Care, Special Care, without External Carer	1,059		
XA04Z	Neonatal Critical Care, Special Care, with External Carer	843		
XA06Z	Neonatal Critical Care, Transportation	1,657		
XB09Z	Paediatric Critical Care, Enhanced Care	1,048		
**HRG code**	**Currency description**	**Day case (£)**		
PX57B	Paediatric, Examination, Follow-up, Special Screening or Other Admissions, with CC Score 1-3	706		
Outpatient care; Source: National schedule of NHS costs - Year 2023/24[14]				
**Service Description**		**Unit Cost (£)**		
Paediatric Service - First Attendance		381		

Paediatric Service – Follow up Attendance	303
Emergency Medicine - Cat 1 Investigation & Treatment	229
Paediatric Dermatology Service	213
Paediatric Audio Vestibular Medicine Service	219
Paediatric Cardiology Service	296
Paediatric Trauma and Orthopaedic Service	217
Ultrasound Scan (20 min+ without contrast)	100
Physiotherapy Service - Face-to-Face	148
Nurse Specialist/Team Leader (Hospital)	56
Paediatric ENT Service	179
Speech and Language Therapy - Face-to-Face	425
Paediatric Surgery Service - Face-to-Face	288
Paediatric Ophthalmology Service	164
Paediatric Neurology Service	555
Paediatric Endocrinology Service	494
Dietetics Service - Face-to-Face	275
Follow-up Phone Consultation	230
Dietetics Service - Phone	168
Paediatric Plastic Surgery Service	197
Paediatric Urology Service	184
Paediatric Clinical Haematology Service	503
CT scan - One Area, No Contrast	105
Paediatric Respiratory Medicine Service	430
Neonatal Critical Care Service	310
Video Physiotherapy - First Attendance	100
Hernia Surgery - Day case	3118
Blood Test - Chemical Pathology	6
Blood Transfusion - Outpatient	433
X-Ray - Plain Film	101
Oxygen Therapy Monitoring	83
Gas Test - Blood/Oximetry	72
MRI Scan - One Area, No Contrast	216
Formula; Source: Prescription cost analysis - Year 2023/24[18]	
**Product Description**	**Unit Cost (£)**
Nutriprem 2 Powder	40.46
Nutriprem 2 Liquid	109.21
Alimentum Powder	37.04
SMA Alfamino Powder	42.97
SMA High Energy Milk	204.12
SMA PRO Gold Prem 2 Powder	47.93
SMA Althera Powder	44.16
Aptamil Pepti 1 Powder	45.36
Aptamil Pepti 2 Powder	67.01
Similac High Energy Liquid	227.95
Aptamil Anti-Reflux Powder	28.25
EleCare Powder	48.83
Infatrini Peptisorb	378.03
Infatrini Liquid	183.93
Neocate LCP Powder	92.84
Nutramigen 1 with LGG Powder	29.6
Nutramigen 2 with LGG Powder	79.61
Nutricia Flavour Modjul Powder - Pineapple	50.68
Nutramigen PurAmino Powder	137.88
Medications; Source: Prescription cost analysis and CBNF - Year 2023/24[18, 19]	

**Medication Description**	**Unit Cost (£)**
Generic Abidec Multivitamin drops	4.74
Aciclovir 200mg Dispersible Tablets	3.61
Omeprazole 10mg Dispersible Gastro-Resistant Tablets	11.27
Amoxicillin 125mg/5ml Oral Suspension SF	2.87
Oxybutynin 2.5mg Tablets	1.33
Iron Sucrose 100mg/5ml Injection	35.37
Aspirin 75mg Dispersible Tablets	0.92
Hydrocortisone 1% / Miconazole 2% cream	3.38
Ipratropium bromide 20micrograms/dose inhaler	5.86
Gaviscon Powder Sachets	15.9
Baclofen 5mg/5ml Oral Solution	14.16
Beclometasone 50micrograms/dose nasal spray	3.05
Benzydamine 0.15% oromucosal spray sugar free	3.74
Salbutamol 100micrograms/dose inhaler	1.9
Calcium gluconate 1g effervescent tablets	34.29
Paracetamol 120mg/5ml oral solution paediatric sugar free	5.89
Clotrimazole 2% Cream	7.33
Captopril 25mg Tablets	1.8
Carob Seed Flour Powder	8.99
Cetraben Ointment	7.48
Chloramphenicol 0.5% eye drops	10.49
Chlorothiazide 25mg/5ml oral suspension	52.03
Chlorphenamine 2mg/5ml oral solution sugar free	5.14
Colecalciferol 2,000units/ml oral drops sugar free	20.42
Sodium feredetate (iron 27.5mg/5ml) oral solution sugar free	14.13
Ciprofloxacin 0.2% eye drops preservative free	24.6
Clarithromycin 125mg/5ml oral suspension	5.32
Beclometasone 50micrograms/dose inhaler CFC free	5
Clotrimazole 2% cream	7.33
Co-amoxiclav 125mg/31mg/5ml oral suspension sugar free	2.61
Colecalciferol 2,000units/ml oral drops sugar free	20.42
Tilactase 50,000units/g oral drops	15.61
Macrogol compound oral powder sachets NPF sugar free	7.82
Colecalciferol 2,000units/ml oral drops sugar free	20.42
Generic Dalivit oral drops	9.59
Hydrocortisone 1% / Miconazole 2% cream	3.38
Ketoconazole 2% cream	5.33
Docusate sodium 5% ear drops	4.78
Generic Kanso DeliMCT CacaoBar	25.22
Enalapril 5mg/5ml oral suspension	32.4
Epaderm ointment	8.71
Erythromycin ethyl succinate 125mg/5ml susp sugar free	10.45
Esomeprazole 10mg gastro-resistant granules sachets	53.98
Clobetasone 0.05% cream	4.21
Ferrous sulfate 125mg/ml oral drops sugar free	99.17
Sodium feredetate (iron 27.5mg/5ml) oral solution sugar free	14.13
Furosemide 40mg/5ml oral solution sugar free	24.12
Fusidic acid 250mg/5ml oral suspension	38.43
Flucloxacillin 125mg/5ml oral solution	4.49
Folic acid 5mg/5ml oral solution sugar free	53.1
Generic Thick & Easy powder	32.5
Omeprazole 10mg/5ml oral solution	11.27
Sod algin 225mg/Mag algin 87.5mg/dose oral	15.9

Gentamicin 20mg/2ml solution for injection vials	89.92
Glucose 75g/300ml oral solution	4.44
Glycerol 40% cream	7.29
HealthAid Babyvit Multivitamin liquid drops	7.53
Heparinoid 0.3% cream	4.98
Hydrocortisone 1% / Miconazole 2% cream	3.38
Ibuprofen 100mg/5ml oral suspension sugar free	9.65
Omeprazole 10mg dispersible gastro-resistant tablets	11.27
Simeticone 40mg/ml oral suspension sugar free	6.14
Carob seed flour powder	8.99
Ipratropium bromide 21micrograms/dose nasal spray	8.06
Levetiracetam 100mg/ml oral solution sugar free	31.75
Lactulose 3.1-3.7g/5ml oral solution	3.38
Lansoprazole 5mg/5ml oral suspension	13.24
Macrogol compound oral powder sachets NPF sugar free	7.82
Mometasone 25microg / Olopatadine 600microg/dose nsl spy	14.24
Montelukast 4mg granules sachets sugar free	7.87
Macrogol compound oral powder sachets NPF sugar free	7.82
Salbutamol 5mg/ml nebuliser liquid	4.91
Phytomenadione 200micrograms/ml oral drops sugar free	18.58
Nystatin 100,000units/g/Chlorhex Hcl 1% / Hydrocort 0.5% crm	11.04
Antazoline 0.5% / Xylometazoline 0.05% eye drops	3.83
Paracetamol 120mg/5ml oral solution paediatric sugar free	5.89
Phenoxymethylpenicillin 125mg/5ml oral solution	12.99
Phenoxymethylpenicillin 125mg/5ml oral solution	12.99
Chlorphenamine 2mg/5ml oral solution sugar free	5.14
Prednisolone	3.6
Propranolol 10mg tablets	2.02
Betamethasone valerate 0.1% cream	3.86
Ranitidine 75mg/5ml oral solution sugar free	12.22
Sodium chloride 300mg/5ml oral solution	13.03
Sodium chloride 0.9% eye dps 0.5ml ud preservative free	20.87
Senna 7.5mg/5ml oral solution	6.33
Sodium bicarbonate 8.4% (1mmol/ml) inj 100ml bottles	78.5
Spironolactone 25mg tablets	1.93
Generic Anusol HC ointment	3.4
Colecalciferol 10,000units/ml oral drops sugar free	13.02
Generic Timodine cream	3.95
Dorzolamide 20mg/ml / Timolol 5mg/ml eye drops	6.22
Trimethoprim 50mg/5ml oral suspension sugar free	7.9
Solgar Vitamin D 400unit capsules	15.65
Phytomenadione 200micrograms/ml oral drops sugar free	18.58
Ascorbic acid powder	13.01
Zeroveen cream	7.06
Magn glycerophos (mag 97.2mg/5ml (4mmol/5ml)) susp	15.27
Vancomycin 500mg powder for concentrate for solution for infusion vials	14.75

Table A2; Full milk feeding vs gradual feeding; NHS and PSS costs for complete case, study period, and cost category (£, 2023–24 prices); Excluding readmission neonatal intensive care data

Cost description[1#]	Treatment group, mean adjusted[2] (SE[3]) cost (£)		Between-group differences (95% CI)	p-value
	Full milk feeding (n=1,029)	Gradual feeding (n=1,023)		
Infants with complete data	N = 512	N = 465		
**Initial admission cost (from randomisation to initial discharge)**				
Neonatal intensive care (days)	2,425.41 (429.56)	3,921.98 (697.56)	-1,496.57 (-2,851.65 to -141.49)	0.030
Neonatal high dependency care (days)	7,235.00 (721.59)	7,549.32 (779.55)	-314.32 (-2.129.54 to 1,500.89)	0.734
Neonatal special care (days)	27,709.83 (767.83)	25,591.78 (728.84)	2,118.05 (723.65 to 3,512.44)	0.003
Special care with primary carer resident or transitional care	1,103.23 (90.05)	1,389.52 (121.43)	-286.28 (-584.09 to 11.52)	0.060
Transfer to different hospital	643.57 (92.58)	533.35 (77.72)	110.22 (-120.24 to 340.69)	0.349
Antibiotic[4]	0.03 (0.00)	0.03 (0.00)	0.00 (-0.01 to 0.01)	0.979
**Total initial admission cost**	**38,393.98 (775.02)**	**39,222.47 (814.26)**	**-828.48 (-2,427.66 to 770.69)**	**0.310**
**Six weeks' corrected age**				
GP visit (surgery)	79.29 (4.82)	77.80 (5.00)	1.40 (-12.22 to 15.01)	0.840
GP visit (home)	0.10 (0.01)	0.08 (0.01)	0.02 (0.00 to 0.05)	0.036
GP phone	68.89 (6.98)	64.85 (6.73)	4.03 (-14.72 to 22.78)	0.673
Practice nurse	14.95 (1.17)	15.01 (1.25)	-0.06 (-3.40 to 3.28)	0.971
Health visitor at surgery	7.25 (0.60)	7.61 (0.67)	-0.36 (-2.12 to 1.39)	0.684
Neonatal nurse (home)	85.47 (21.27)	78.23 (18.26)	7.24 (-15.88 to 30.36)	0.540
Health visitor (home)	72.53 (2.47)	68.17 (2.46)	4.36 (-2.48 to 11.19)	0.212
Walk-in centre	65.67 (9.64)	56.55 (8.94)	9.11 (-16.91 to 35.13)	0.492
Visit of any other health professionals - community based	20.87 (5.70)	34.77 (10.12)	-13.89 (-36.69 to 8.91)	0.232
**Community cost**	**416.45 (15.60)**	**396.75 (15.68)**	**19.71 (-19.69 to 59.10)**	**0.327**
Paediatric ward	523.43 (122.16)	240.04 (58.34)	283.40 (20.73 to 546.06)	0.034
Day case	273.20 (38.09)	294.89 (43.60)	-21.68 (-133.96 to 90.59)	0.705
Outpatient clinic	412.04 (22.51)	415.11 (24.15)	-3.07 (-66.81 to 60.67)	0.925
Accident and emergency (A&E) department	86.61 (7.92)	92.98 (9.07)	-6.37 (-30.00 to 17.26)	0.597
Visit of any other health professionals - hospital based	100.91 (20.75)	58.83 (13.06)	42.08 (-5.36 to 89.52)	0.082

Procedures	16.56 (12.05)	5.03 (2.67)	11.53 (-11.71 to 34.78)	0.3317
**Total hospital cost**	**1,363.55 (141.37)**	**1,134.54 (123.67)**	**229.01 (-92.39 to 550.43)**	**0.163**
Formula	123.04 (16.32)	145.24 (19.68)	-22.20 (-64.28 to 19.89)	0.301
Medications	20.56 (1.86)	21.93 (2.05)	-1.37 (-6.34 to 3.60)	0.588
**Six-week follow-up**	**1,773.09 (92.33)**	**1,701.24 (93.30)**	**71.84 (-150.40 to 294.09)**	**0.526**
**Total cost**	**40,266.44 (810.22)**	**40,875.73 (810.22)**	**-609.29 (-2,252.22 to 1,033.63)**	**0.467**

[#] Note costs have changed slightly due to random effects

[1] Healthcare costs are capped and reported with a maximum of 112 days (reflecting the study follow-up period).

[2] Adjusted for gestational age, birthweight centile, and intravenous fluids; random effects account for clustering by site and mother (to capture multiple births).

[3] SE, Standard error
